# Spatiotemporal neurodynamics of automatic temporal expectancy in 9-month old infants

**DOI:** 10.1038/srep36525

**Published:** 2016-11-04

**Authors:** Giovanni Mento, Eloisa Valenza

**Affiliations:** 1 Department of General Psychology, University of Padua. Via Venezia, 8, 35131, Padova (PD), Italy; 2 High-Density EEG Interdepartmental lab (LIGA). University of Padua, Italy; 3 Department of Developmental Psychology and Socialization, University of Padua. Via Venezia, 8, 35131, Padova (PD), Italy; 4 Centro di Neuroscienze Cognitive. University of Padova, Italy

## Abstract

Anticipating events occurrence (Temporal Expectancy) is a crucial capacity for survival. Yet, there is little evidence about the presence of cortical anticipatory activity from infancy. In this study we recorded the High-density electrophysiological activity in 9 month-old infants and adults undergoing an audio-visual S1–S2 paradigm simulating a lifelike “Peekaboo” game inducing automatic temporal expectancy of smiling faces. The results indicate in the S2-preceding Contingent Negative Variation (CNV) an early electrophysiological signature of expectancy-based anticipatory cortical activity. Moreover, the progressive CNV amplitude increasing across the task suggested that implicit temporal rule learning is at the basis of expectancy building-up over time. Cortical source reconstruction suggested a common CNV generator between adults and infants in the right prefrontal cortex. The decrease in the activity of this area across the task (time-on-task effect) further implied an early, core role of this region in implicit temporal rule learning. By contrast, a time-on-task activity boost was found in the supplementary motor area (SMA) in adults and in the temporoparietal regions in infants. Altogether, our findings suggest that the capacity of the human brain to translate temporal predictions into anticipatory neural activity emerges ontogenetically early, although the underlying spatiotemporal cortical dynamics change across development.

The ability to anticipate the onset of relevant stimuli, also known as Temporal Expectancy, is a fundamental skill because it allows us to orient our attention in time and consequently adapt our behaviour towards upcoming events^1^,^2^. For example, the ability to anticipate a task-relevant signal translates into significant behavioural benefits in terms of speed and accuracy, both in adults^1^ and school-aged children^3^,^4^. In neurophysiological terms, temporal expectancy implies the possibility of implementing anticipatory neural mechanisms towards predictable *vs.* unpredictable upcoming stimuli. Specifically, the Contingent Negative Variation (CNV) has been proposed as a reliable Event-related Potential (ERP) signature directly depicting the online dynamic of temporal expectancy building up over time, both in adults^5^,^6^,^7^,^8^,^9^ and school-aged children^10^.

Crucially, the ontogenesis of the neurocognitive mechanisms underlying the ability to establish and implement expectancy over time has slipped out of core attention for many years and this lack of developmental evidence is even more surprising if considering that from birth any sensory and motor experience we undergo is nestled in the temporal dimension. For example, our first affective and social interactions with conspecifics are featured by precise temporal structures and dynamics^11^, suggesting that a ‘sense of time’ is ontogenetically early. In line with this argument, researchers demonstrated that the ability to perceive and represent the temporal information is already present in the first months of life. For example, few month-old infants are able to learn the temporal intervals between events as well as the durations associated with events, as demonstrated by decelerated heart rate to an omitted timed sensorial event^12^,^13^. Interestingly, some of the same mechanisms as adults for detecting deviations in the timing of stimulus events have been reported in 10-month-old infants^14^. Moreover, 3-month-old infants can learn the spatiotemporal pattern of visual presentations and use this knowledge to make anticipatory eye movements before the onset of an expected sensorial stimulus^15^. Yet, if and how early the ability to implicitly use this temporal information can translate into specific, cortical anticipatory mechanisms towards future events has been much less investigated. Some studies have provided evidence that infants as young as 3-months can implement saccade-preceding ERPs towards lateralized, expected stimuli^16^. Yet, it is still unclear whether this anticipatory activity is specific to motor planning/preparation or is more general in nature, being instantiated whenever the sensory environment is temporally structured and regardless of the need to implement stimulus-driven actions, like in the case of passive, central fixation paradigms^5^.

In this ERP study we tried to address the specific question as to whether task-independent, anticipatory cortical activity preceding expected stimuli is operational at early age. To such purpose, we collected the high-spatial resolution EEG signal in both 9-month-old infants and adults with an experimental paradigm purposely designed to automatically induce temporal expectancy of socially-relevant stimuli. The age of 9 months was purposely chosen because the ability to predict the occurrence of audiovisual events is stable at this age^17^,^18^.

We adapted the temporal oddball passive paradigm (TOP) from our previous study on adults^6^, with the explicit purpose of simulating a lifelike “Peekaboo” game (Fig. 1). The advantage of this paradigm is to automatically and progressively induce the implicit learning of regular temporal contingencies in a free looking context and in the absence of instruction comprehension and/or motor responses^5^. The use of the same paradigm in adults and infants allowed us to qualitatively compare adults’ and infants’ neural anticipatory activity with the purpose of understanding whether (1) anticipatory neural mechanisms instantiated by temporal expectancy are already operating early in life and (2) these relay on similar or different spatiotemporal dynamics as compared to adults.

## Results

Inspection of both infants and adults’ Grand-averaged ISI-related ERP revealed evidence of clear electrophysiological activity consisting of a slow, sustained CNV-like wave (hereafter called simply CNV) arising early soon after the onset of the ISI and going on until S2 presentation. A one-sample cluster-based permutation analysis revealed in both groups two distinct clusters of spatiotemporally adjacent electrodes whose signal amplitude was statistically different (t > ± 2.14; Family Wise Error corrected p < 0.05) from the zero baseline for a temporal window extending from about 350 ms to the end of the ISI (Fig. 2a). Remarkably, while in both groups the ERP activity exhibited similar temporal dynamic and morphological pattern, visual inspection of the spatial maps indicated a different scalp distribution in adults compared to infants. In fact, while in adults the CNV was spatially expressed as an anterior-positive/posterior-negative dipole, in infants the positive counterpart of this wave was localized more centrally, suggesting that at least partially different neural generators in adults and infants may underpin the CNV.

The analysis of the time-on-task effects, defined as the dynamic changes in anticipatory ERP activity across the task^5^,^6^, revealed a significant modulation of the CNV amplitude. In particular, in both groups we identified two distinct clusters of electrodes whose amplitude was significantly modulated from the early to the late phase of the task. This finding was supported by the cluster-based permutation analysis (t > ± 2.14; Family Wise Error corrected p < 0.05).

Specifically, ERP activity in the posterior cluster became more negative, while the anterior/central one more positive, as the task progressed. Interestingly, in the posterior cluster this effect showed different latency ranges in the two groups, since in adults it emerged 170 ms after ISI onset and extended over the whole epoch. By contrast, the time-on-task effect in infants took place 400 ms after ISI onset.

In other words, in both groups anticipatory neural activity became globally larger as the temporal contingency between S1 and S2 in the delayed condition was implicitly discovered and learned.

Importantly, when looking at single subjects (Fig. 3), we found that this effect was present in all participants, as confirmed by an additional paired t-test contrasting the mean early vs. late CNV amplitude for both adults (Anterior Cluster: t(13) = −9.88; p < 0.001; Posterior cluster: t(13) = 6.95; p < 0.001) and Infants (Central Cluster: t(11) = −6.67; p < 0.001; Posterior cluster: t(11) = 8.34; p < 0.001).

### Brain Source Reconstruction

As shown in Fig. 4, our exploratory brain source reconstruction identified both common and distinct areas between adults (Fig. 4a) and infants (Fig. 4b) being activated over the whole CNV interval. Common areas included the right prefrontal cortex and in particular the inferior and the middle frontal gyrus. Infants also exhibited a strong activity over the temporoparietal areas, with a larger activity on the right hemisphere. Specifically, the temporoparietal activity extended over the inferior (IPC) and the superior (SPC) parietal cortex, the inferior (ITG), the middle (MTG) and the superior (STG) temporal lobe. By contrast, adults showed a strong activation of the Supplementary Motor Area (SMA). The time course of the cortical activations is shown in Supplementary Video S1. Remarkably, in both groups we found an opposite time-on-task pattern differentiating the anterior and the posterior areas (Fig. 4c). Specifically, the r-MFG exhibited a larger activity in the early phase of the task, while the age-specific cortical areas identified were more activated in the late phase of the task.

## Discussion

In the present study we report electrophysiological evidence of anticipatory neural activity towards temporally expected events in 9-month-old infants. Specifically, we show that such activity relays on an adult-like CNV, extending the reliability of such component as an hallmark of temporal expectancy from adult^6^,^7^,^8^,^9^ to the infant ERP literature.

Adult studies suggested that the CNV reflects a reliable ERP signature directly depicting the online dynamic build up of temporal expectancy^19^. In this framework, the presence of an adult-like CNV in 9-month-old infants might represent an early electrophysiological signature of anticipatory neural activity reflecting a ‘core’ expectancy process dissociable from motor preparatory mechanisms, such as those related to oculo-motor control in spatial attention paradigms^16^,^20^. As far as we know, only one functional Near Infrared Spectroscopy (fNIRS) study by Nakano *et al*.^21^ purposely investigated expectancy-based anticipatory neural activity in the developing brain, reporting fronto-temporoparietal hemodynamic activity preceding stimulus presentation in sleeping 3-month-old infants. Although alertness may constitute a potential confound in the study of Nakano and colleagues^22^,^23^, our findings are consistent with it to the extent that the CNV we report here can be interpreted as the electrophysiological counterpart of the hemodynamic anticipatory activity. Indeed, we show electrophysiological evidence converging to hemodynamic findings in suggesting that expectation-related anticipatory activity is an inherent, early property of the cortex, as previously proposed for expectancy-related feedback in expectancy-violation paradigm^24^,^25^. The interpretation that the CNV reflects a specific anticipatory signature of temporal expectancy rather than other physiological effects such as arousal fluctuations against the zero baseline preceding the onset of the delayed S2 was further corroborated by the presence of time-on-task effects. These consisted of larger CNV amplitude as the task progressed both in adults (in line with our previous findings^6^) and infants. On the one hand, this finding provides electrophysiological support to behavioural evidence that implicit learning is already operational in young infants^26^. On the other hand, it might suggest a possible early mechanism at the basis of expectancy building-up as the temporal uncertainty towards upcoming events is reduced. As a limitation of the study, the relative small number of artifact-free trials accepted for the delayed condition did not allow to further split the data into more blocks, that would have allowed to further test the linearity of the implicit learning effect across the task.

The CNV cortical source reconstruction revealed that expectancy-based neural anticipatory activity in infants is mainly supported by the engagement of both common and distinct networks compared to adults. Specifically, we found a common source in adults and infants in the right prefrontal cortex, which might suggest that this area may play an ontogenetically early role in implicit learning processes at the core of automatic anticipatory behaviour. In this sense, time-on-task decreases in right prefrontal cortex activity may indicate that this area is especially engaged in the initial phase of the implicit learning of the S1–S2 contingency in the delayed trials. This account is consistent with the observation that in adults prefrontal regions have been shown to be required more when learning a task or acquiring a new skill, than once it is acquired^27^.

Additional CNV generators were found in other cortical areas, which were nevertheless age-dependent. This finding is not surprising given the considerable differences in neural organization, connectivity, and efficiency between infant and adult brains^28^,^29^. In particular, adults showed activity in the SMA, while infants exhibited a wide and strong activity in the posterior regions including parietal and temporal areas. A possible explanation for the SMA recruitment in adults might take into account the functional link between such region and the motor/premotor areas responsible for timed motor preparation and execution^6^,^30^. Hence, an increased time-on-task SMA activity in adults might suggest a progressive functional tuning of this area with the S1–S2 interval as far as the temporal structure of the delayed trials is implicitly discovered and learnt. Indeed, the expectation of an event may automatically instantiate temporal tuning for preparing a possible motor response to that event even in the absence of explicit motor task-demand, as we previously reported^6^. By contrast, the SMA may be not yet attuned to temporal expectancy at 9 months of age given that timed motor behaviour triggered by predictable events is a competence emerging at a later age^31^. As the child motor repertoire enriches, it might be assumed that this area incorporates a more complex functional role directly related to motor preparation induced by stimuli expectancy. In other words, this structure might become functionally specialised to combine motor and timing processes across development. This hypothesis is in line with the ‘neuronal recycling’ account that action circuits are progressively engaged across development in order to build up representations of time^32^,^33^. This view implies that the ontogenetic roots of our ‘sense of time’ could be originally abstract in nature but become inextricably tied to action across development. In line with this account, in a recent study we found an expectancy-related SMA involvement at 8 years of age^10^.

Concerning the temporoparietal source of CNV, which was identified only in infants, a possible explanation may be that such regions reflect stimulus-preceding anticipatory activity underlying perceptual processes. As previously suggested by Nakano *et al*.^21^, the implicit learning of the temporal contingency between two stimuli may induce an endogenous temporal orienting of attentional, resulting in the prioritizing of the perceptual processing of the expected stimulus, in line to adult studies^7^,^9^,^34^. Therefore, it is possible that, as long as the prefrontal cortex has learned the S1–S2 temporal contingency in the delayed trials, this knowledge is progressively transferred to posterior cortical areas deputed to sensorial processing of the expected stimuli. From a neurophysiological point of view, this mechanism would translate into a decrease of prefrontal activity and in a higher recruitment of the cortical activity in posterior region, as we actually observed. Conversely, the time-on-task decrease of posterior anticipatory cortical activity in adults may be explained by the absence of either motor or perceptual S2-related overt task demand, which may have caused a progressive neural habituation of perceptual areas to repetitive stimuli. In fact, it is important to consider that, unlike adults, the social nature of the expected stimulus (smiling female faces) may have been sufficiently attractive for infants to keep paying increasing attention on it over the whole task. Indeed, faces are the most salient and frequent visual stimulus category available in infants’ environment and familiar stimuli. As such, they enhance infants’ ability to detect and generalize abstract rules^18^,^26^,^35^,^36^. It is also noteworthy to consider that the posterior cortical activity in infants spread over adjacent but possibly functionally distinct cortical areas, suggesting that the CNV may embody functionally distinct anticipatory processes. These may include the implicit timing of sensory events, which has been consistently associated to the inferior parietal cortex^32^,^37^,^38^ or the anticipation of auditory stimuli, which may involve the temporal areas^39^. It is also possible that the use of socially-relevant stimuli like faces may have resulted in a strong pre-activation of the posterior superior temporal sulcus as well as of the surrounding regions, as already shown in six- to seven-month-old infants^40^. However, when interpreting these findings it is critical to bear in mind that the interpretation of source reconstruction in infants requires caution, due to the limitations of the EEG in spatial accuracy^41^ as well as to the fact that we did not have single-subject head models or three-dimensional coordinates about the actual electrode scalp positions, which would have improved cortical source localization accuracy. Moreover, the segregation of the cortical anticipatory activity into functionally distinct areas is beyond the aim of the present study, that was to demonstrate that the expectancy-related cortical anticipatory activity is an ontogenetically early property of the human neurocognitive system that nevertheless imply partially different networks as compared to adults. Further studies using high-spatial resolution neuroimaging technics such as the fNIRS would be more appropriate to address the issue of whether functionally distinct cortical areas are recruited when anticipating expected stimuli.

Notwithstanding these limitations, we can be reasonably confident in our findings because: (1) we used age-appropriated adjustments, including forward modelling and age-appropriated standard anatomical templates, in line with literature^24^,^42^ and (2) they confirm previous evidence on the mapping of hemodynamic anticipatory cortical activity in infants^21^.

Altogether our findings suggest that the ability to generate expectancy-based anticipatory brain activity is operational early across development, although the neural networks underlying such ability change across development as a consequence of motor experience and neural maturation. From a theoretical perspective, the present study adds new knowledge on the understanding of the human brain as a complex machinery biologically predisposed to construct temporal predictions about external events^24^,^25^. More specifically, our findings might suggest an ontogenetic continuum between infants and adults in the mechanisms at the basis of expectation-based anticipatory behaviour. In fact, notwithstanding the bulk of structural and functional changes occurring during development, the capacity of the human brain to extrapolate environmental sensory regularities and consequently shape its cortical activity to the structure of the environment may constitute a basic, early property of the human neural architecture. In this respect, our findings go a little step further previous studies by identifying in the CNV a possible developmental electrophysiological anticipatory signature of temporal expectancy building up over time. As well, the CNV time-on-task pattern may reflect a global, extensive modulatory mechanism operating early in life and based on the progressive reduction of temporal uncertainty over time, with the final goal of allowing humans to create an adaptive, internal model of the environment.

Finally, from a broader viewpoint, our findings might have future implications for clinical policies: there is increasing evidence that the ability of generate temporal prediction and consequently orienting attention in time may be compromised in some major pediatric neuropsychiatric disorders including Attention Deficit/Hyperactivity Disorder (ADHD) and Autistic Spectrum Disorders^43^. In this vein, the possibility of setting up an infant-friendly experimental setting in order to obtain a reliable ERP marker of anticipatory neural activity may facilitate an early distinction between typical and atypical development.

## Methods

### Participants

Fifteen healthy infants were included (mean age 283 days; range 252–333 days, SD 23.9 days; 5 females). Data from additional infants were rejected because of fussiness (n = 3) or excessive movements (n = 3). The adult group included fourteen healthy University students (mean age 25 years; range 20–32 years, SD 3.2 years; 9 females). All children’s parents and adult participants signed an informed written consent. All experimental methods had ethical approval from Research Ethics Committee of the School of Psychology of the University of Padua (prot. N. 1179). The methods were carried out in accordance with the approved guidelines and regulations.

### Stimuli

Infants were presented with synchronized audio–visual sequences which simulating a “Peekaboo” animation. Visual stimuli consisted of pictures of real female faces, which could be covered by hands or barefaced and smiling. A total of four different identities were randomly delivered after being matched for low-level physical features. Auditory stimuli were digitized samples of the notes C_4_, E_4_, G_4_ and C_5_ played by different musical instruments (i.e., trumpet, oboe, saxophone and piano) freely available from the IOWA University website (http://theremin.music.uiowa.edu/). All auditory stimuli were matched for low-level physical features.

### Experimental Design

**Figure 1:**
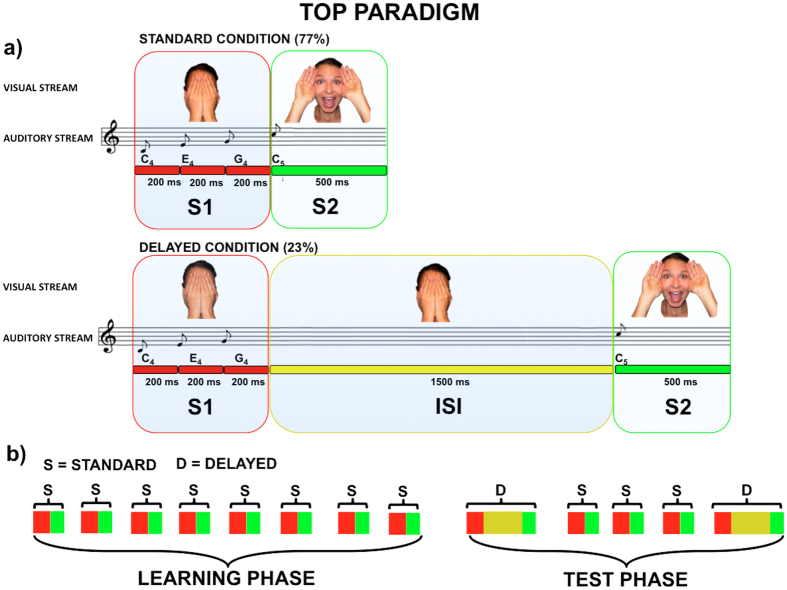
Temporal Oddball Passive paradigm (TOP). (**a**) Both adults and 9-month-old infants are passively presented with a temporal oddball paradigm^6^ simulating a lifelike “Peekaboo” animation game. In the standard condition the simultaneous and equally-long presentation of a triplet of notes (C_4_, E_4_ and G_4_, 200 ms each) together with the picture of a 600-ms covered face constituted the S1 stimulus (red square). S1 was followed by the S2 stimulus, which consisted of the synchronized delivery of a fourth note (C_5_) together with a picture displaying a female smiling face (green square), both lasting 500 ms on the screen. In the standard condition (77%), the S1–S2 Stimulus Onset Asynchrony was fixed (600 ms), forming a seamless audio-visual sequence. In the delayed condition (23%), S1 was followed by a fixed 1,500-ms ISI (yellow square). The HD-ERP activity elicited by ISI onset in the delayed condition was extracted and analysed in order to explore the presence of sustained S2-anticipatory cortical activity. (**b**) An extract from a typical block sequence in which standard (S) and delayed (D) trials alternate, each one including S1 and S2.

**Figure 2:**
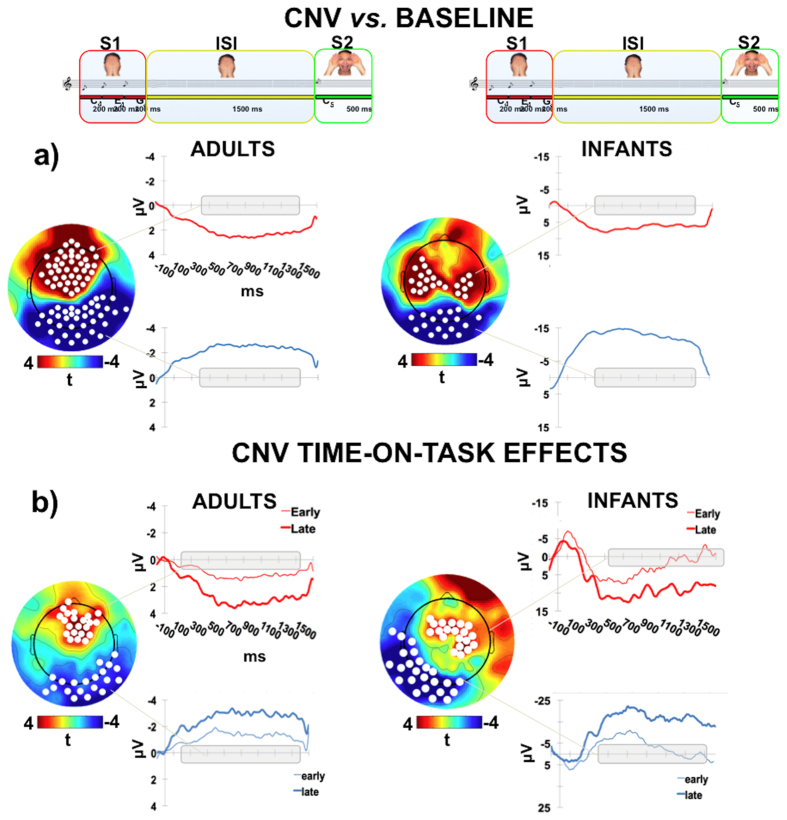
ISI-related ERP activity. (**a**) The plots of the t-score scalp maps show the spatial distribution of the electrodes (white dots) exceeding the critical t-score threshold for statistical significance at the one-sample cluster-based permutation test. In both adults and infants significant electrodes are grouped into distinct spatiotemporal clusters showing positive (in red) or negative (in blue) amplitude difference against the zero baseline. The waveforms on the right of each map represent the grand-average ERP time course for each cluster. The grey bar on the x-axis represents the temporal extent of statistical significance for each cluster. (**b**) The t-score scalp maps show the distribution of the electrodes sensitive to time-on-task effects. In particular, in red are represented scalp regions showing more positive ERP amplitude from the early to the late phase of the task, while in blue are represented the regions showing more negative ERP amplitude over time. The waveforms on the right of each map display the early (thin line) *vs.* late (thick line) ERP comparison. The grey bar on the x-axis represents the temporal extent of the time-on-task statistical effect for each cluster.

**Figure 3:**
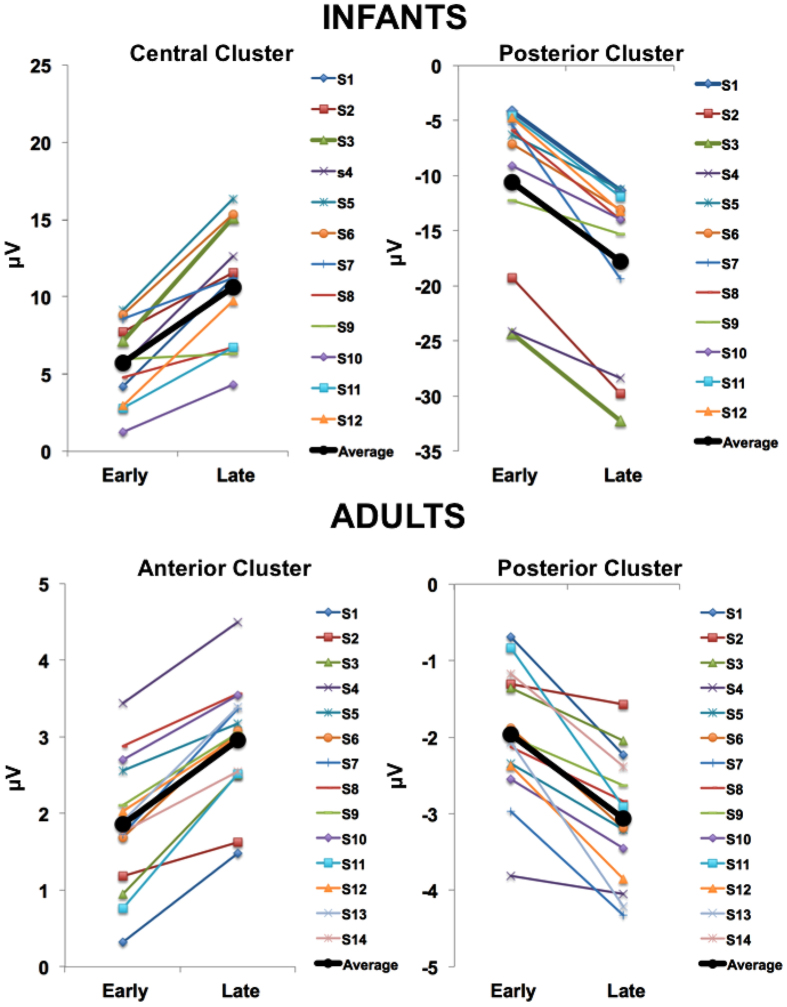
Single-subject CNV time-on-task effects. Each line represents a single subject. The markers plot the CNV amplitude modulation between the early and the late phase of the task for adults (upper panel) and infants (lower panel). The CNV amplitude calculated as the average of all significant electrodes in each cluster in the time window of 400 to 1,500 ms from ISI onset.

**Figure 4:**
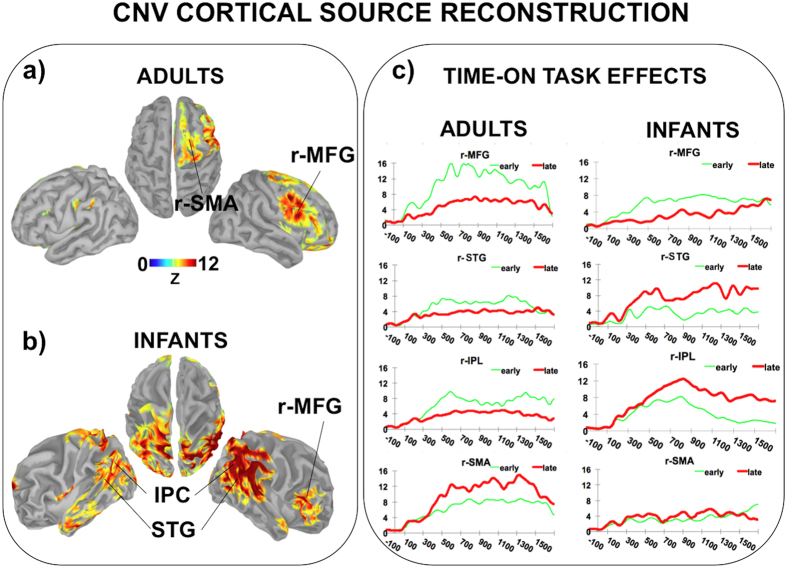
CNV cortical source reconstruction. The maps on the left display the reconstruction of the averaged cortical activity relative to the whole ISI-related CNV in adults (**a**) and infants (**b**). A common cortical activation between them can be identified in the right prefrontal cortex, mainly including the middle frontal gyrus (r- MTG). Moreover, adults exhibit additional activity in the right supplementary motor area (r-SMA) while infants show a strong activation of the posterior parte of the brain, spreading over parietal areas, including inferior (IPC) and superior (SPC) parietal cortex and temporal areas, including inferior (ITG), middle (MTG) and superior (STG) temporal gyrus. The panels on the right (**c**) show the comparison between the time course of the averaged normalized activity in the early (thin line) *vs.* the late (thick line) phase of the task (time-on-task effects) relatively to the most relevant cortical regions implied in adult and infant anticipatory ERP activity.

The experimental design is illustrated in Fig. 1. Each trial began with the presentation of a face covered by hands that appeared on the centre of the screen for 600 ms. The presentation of the visual stimulus was matched with the onset of a triplet of notes, each of them lasted 200 ms. This first audio-video sequence was defined as S1.

Different harmonic sequences of notes (i.e., ascending or monotonic sequences) were associated to the presentation of the covered face. However, since exploratory analysis did not yield significant effects of harmonic sequence on S2-anticipatory ERP activity, these were averaged together. To maintain infants’ attention, the identity of the faces and the type of musical instrument were changed randomly trial-by-trial. S1 was seamlessly followed by the onset of the same face that appeared uncovered and smiling and that was matched with a fourth C_5_ single note. The presentation of both the uncovered face and the C_5_ note lasted 500 ms and was defined as S2. Globally, the whole sequence was intended to simulate a “Peekaboo” animation in which a woman discovered her face following a regular rhythm paced by the auditory sequence of note.

To elicit a sustained, anticipatory ERP activity we manipulated the S1–S2 temporal contingency, leading to a standard and a delayed condition.

In the standard condition, corresponding to the 77% of the trials, no intervals were embedded between S1 and S2. In other words, there was no S1–S2 ISI. By contrast, in the delayed condition, corresponding to the 23% of the trials, a fixed 1,500 ms interval was embedded between S1 and S2. In this condition, which was randomly delivered in order to avoid anticipatory biases, the temporal contingency was violated generating in the participants a temporal expectancy about the time of the onset of the uncovered face. Noteworthy, no other filled or empty intervals were embedded between S1 and S2 in any case. In other words, S2 could appear either immediately after S1 offset (standard trials) or after a fixed 1,500 ms interval from it (delayed trials).

The Inter Trial Interval (ITI) was randomly varied between 500 and 1,500 ms to avoid to induce temporal expectancy toward the beginning of the next trial.

A total of four blocks were delivered, each lasting about 2 min. As shown in Fig. 1b, for each block there was an initial learning phase, with the first 8 trials (excluded from analysis) belonging to the standard condition. This phase was followed by a test phase of 70 trials, in which 77% of the trials (54) were standard but 23% (16) belonged to the delayed condition. The delayed trials were always followed by at least three standard trials, the first of which was not included in the analyses. The experimental session was stopped if the infant became fussy or after the four blocks. As a limitation of all the studies using an oddball probabilistic distribution, the relative small number of deviant trials in the present study (i.e., here defined as the ‘delayed trials’) did not allow us to further manipulate the experimental paradigm by including additional conditions.

### EEG recording and signal processing

The EEG was continuously recorded and amplified using a geodesic EEG system (EGI GES-300), through a pre-cabled high-density 128-channel HydroCel Geodesic Sensor Net (HCGSN-128) and referenced to the vertex. The sampling rate was 500 Hz. The impedance was maintained below 50 KΩ for each sensor. All EEG recordings were processed offline using the MATLAB toolbox EEGLAB^44^. A band-pass filtered between 0.1 and 20 Hz was applied. During the whole session, the infants’ gaze was monitored via a camera. This allowed a trained experimenter to manually display on the screen attention-getter audio-visual stimuli (cartoon scenes) to re-capture infants attention on the screen whenever they looked away from it but also to off-line reject from the analysis the trials in which the visual stimuli were disregarded. To depict the temporal dynamics of the anticipatory brain activity, the ISI-related ERP signal (−100 to 1,700 ms) elicited in the delayed trials was extracted. After interpolating bad channels, data were subjected to Independent Component Analysis (ICA^41^,^45^) to isolate highly characteristic artifacts, including eye blinks and movements. Artifactual components were discarded, and the remaining ones were projected back to the electrode space to obtain cleaner EEG epochs. The epochs containing excessive noise or drift (±100 and ±150 μV at any electrode for adults and infants, respectively) were further rejected. Data were then re-referenced to the average of all electrodes and the signal was aligned to the baseline. Subject average and grand average ERPs were generated for each electrode site and condition. A total of 56 ± 6 (range: 43–63) and 28 ± 8 (range: 16–45) artifact-free delayed epochs were accepted for adults and infants, respectively. Time-on-task effects were investigated by splitting the whole dataset into two clusters of data, defined as the ‘early’ and the ‘late’ phase of the task. This clustering was done a priori before trial rejection in order to target the same learning phase for each participant and to avoid possible biases in dataset splitting, which may potentially derive from clustering data after trial rejection. In order to obtain a reliable within-subject signal-to-noise ratio (deHaan, 2007), we included only participants showing at least twelve artifact-free trials per phase. All adult participants were included in the analysis. Whereas, two infants over fifteen were excluded since they did not have a sufficient number of collected trials per phase. A further infant was discarded from time-on-task analysis since he showed as few as 10 trials per phase after trial rejection.

The final number of participants included in the time-on-task analysis was 12 over the original 15 infants with, a mean number of 27 ± 2 and 16 ± 3 trials per condition for adults and infants, respectively. Respect to the originally collected trials, the amount of artifact-free trial per phase was comparable in the two groups (p = 0.41), corresponding to the 85% ± 10 and to the 86% ± 10 for adults and infants, respectively. This ruled out the possibility that the time-on-task effects may be biased by a between-group imbalance in trial sampling.

### Statistical analysis

To test for the presence of a sustained, anticipatory neural activity significantly different from noise (i.e., no anticipatory activity hypothesis), in both adults and infants the whole-epoch ISI-related activity was contrasted against the zero baseline. A one-sample, two-tailed cluster-based permutation test based on the cluster mass statistic^46^,^47^ using the original data and 2,500 random within-participant permutations of the data was used. This non-parametric statistical approach allowed us to test the specific hypothesis that the significant differences against the zero-baseline are consistent in space (only neighbouring electrodes showing significant effects are included into a single cluster) as well as in time (the direction of the effects must be the same for the whole epoch considered). In other words, spatiotemporally sparse or inconsistent, although large effects are ignored, capitalizing on the fact that expectancy-related anticipatory cortical activity engages spatiotemporally stable neural networks in both adults and infants. Electrodes within approximately 1.5 cm of one another were considered spatial neighbours and adjacent time points were considered temporal neighbours. All pairs whose t-values were larger than a pre-determined threshold of ±2.14 (corresponding to a Family Wise Error corrected alpha value of 0.05) were considered significant. To statistically test for the presence of time-on task effects the same analysis was applied but this time contrasting the early *vs.* the late phase ERP activity within-group.

### Brain Source Reconstruction

Although a direct comparison of brain source reconstruction across different ages is made difficult by the presence of brain structural differences, including a different brain volume conduction, scalp thickness and dipole orientation (Reynolds and Richards, 2009), the use of a high-density electrode array (i.e., ≥128), together with other age-appropriate adjustments (see below), allowed us to cautiously attempt a qualitative comparison of the cortical areas generating the CNV between infants and adults. To reconstruct cortical sources we applied the same procedures described in literature for adults^6^,^7^ and infants^24^,^48^. In particular, the cortical sources of either the whole and the early- vs. late-phase ERP grand-averages were reconstructed using Brainstorm^49^. Age-appropriate template for adults (MNI templates) and infants^42^ were used after warping the geometry of the EEG sensor net to fit the head mesh. A distributed model of 15,028 current dipoles, whose locations were constrained to the cortex, was applied. The EEG forward solution was computed using an overlapping 3-spheres model^50^ after adjusting head radius and tissue conductivity according to age. Cortical current maps were computed from the EEG time series using the weighted minimum-norm estimation (wMNE) algorithm^51^. The wMNE was constrained to the cortex. A 0.5 order depth weighting was used with a maximal amount of 10. The covariance matrix was assumed to be independent across EEG sensors, with fixed variance computed from pre-stimulus recordings. Source activity was normalized to the baseline. The absolute values of the Z scores were then averaged across participants.

The cortical-ROIs were identified according to the anatomical Tzourio-Mazoyer atlas^52^ adapted for cortical space solution. Finally, to more accurately depict the time course of the activation of the main cortical ROIs identified, we used the scout analysis tool in Brainstorm. A scout represents a region of interest (ROI) in the available source space consisting of a subset of dipoles defined on the cortex surface or the head volume. Once identified the main scouts it is possible to use the dedicated analysis tool to plot and compare their activation time course between different experimental conditions.

## Additional Information

**How to cite this article**: Mento, G. and Valenza, E. Spatiotemporal neurodynamics of automatic temporal expectancy in 9-month old infants. *Sci. Rep.* **6**, 36525; doi: 10.1038/srep36525 (2016).

**Publisher’s note:** Springer Nature remains neutral with regard to jurisdictional claims in published maps and institutional affiliations.

## Supplementary Material

Supplementary Video S1

Supplementary Information
