# Supplementary material for: Spatiotemporal neurodynamics of automatic temporal expectancy in 9-month old infants
Source: Sci Rep. 2016 Nov 4;6:36525. doi: 10.1038/srep36525 (PMC5109914; doi:10.1038/srep36525)
Supplement: Supplementary Information [file srep36525-s2.doc]

**Supplementary Information**

**Spatiotemporal neurodynamics of automatic temporal expectancy in 9-month old infants.**

**Authors: Giovanni Mento^1,2*^ and Eloisa Valenza^2,3,4^**

^1^ Department of General Psychology, University of Padua.Via Venezia, 8, 35131, Padova (PD), Italy.

^2^ High-Density EEG Interdepartmental lab (LIGA). University of Padua, Italy

^3^Department of Developmental Psychology and Socialization, University of Padua. Via Venezia, 8, 35131, Padova (PD), Italy.

^4^ Centro di Neuroscienze Cognitive. University of Padova, Italy.

*= Corresponding Author. Giovanni Mento, PhD. Department of General Psychology, University of Padua, Italy Via Venezia, 8, 35131, Padova (PD), Italy. Tel.: +39 (0) 49 827 6149; Fax: +39 (0) 49 827 6600;

Email: giovanni.mento@unipd.it

**Supplementary file legend.**

**S1 Movie. CNV cortical source reconstruction over time.**

The movie shows the time course of the CNV cortical source reconstruction (-200 to 1500 ms from ISI onset) for infants (top brain, left and right view) and adults (down brain, left and right view).
